# Human Papillomavirus 16, 18, 31 and 45 viral load, integration and methylation status stratified by cervical disease stage

**DOI:** 10.1186/1471-2407-14-384

**Published:** 2014-05-30

**Authors:** Luigi Marongiu, Anna Godi, John V Parry, Simon Beddows

**Affiliations:** 1 Virus Reference Department, Public Health England, 61 Colindale Avenue, London NW9 5EQ, U.K

**Keywords:** Human papillomavirus, Cervical cancer, Genotypes, Viral load, Methylation, Integration

## Abstract

**Background:**

Persistent infection with oncogenic Human Papillomavirus (HPV) is associated with the development of cervical cancer with each genotype differing in their relative contribution to the prevalence of cervical disease. HPV DNA testing offers improved sensitivity over cytology testing alone but is accompanied by a generally low specificity. Potential molecular markers of cervical disease include type-specific viral load (VL), integration of HPV DNA into the host genome and methylation of the HPV genome. The aim of this study was to evaluate the relationship between HPV type-specific viral load, integration and methylation status and cervical disease stage in samples harboring HPV16, HPV18, HPV31 or HPV45.

**Methods:**

Samples singly infected with HPV16 (n = 226), HPV18 (n = 32), HPV31 (n = 75) or HPV45 (n = 29) were selected from a cohort of 4,719 women attending cervical screening in England. Viral load and integration status were determined by real-time PCR while 3’L1-URR methylation status was determined by pyrosequencing or sequencing of multiple clones derived from each sample.

**Results:**

Viral load could differentiate between normal and abnormal cytology with a sensitivity of 75% and a specificity of 80% (odds ratio [OR] 12.4, 95% CI 6.2–26.1; *p* < 0.001) with some variation between genotypes. Viral integration was poorly associated with cervical disease. Few samples had fully integrated genomes and these could be found throughout the course of disease. Overall, integration status could distinguish between normal and abnormal cytology with a sensitivity of 72% and a specificity of 50% (OR 2.6, 95% CI 1.0–6.8; *p* = 0.054). Methylation levels were able to differentiate normal and low grade cytology from high grade cytology with a sensitivity of 64% and a specificity of 82% (OR 8.2, 95% CI 3.8–18.0; *p* < 0.001). However, methylation varied widely between genotypes with HPV18 and HPV45 exhibiting a broader degree and higher magnitude of methylated CpG sites than HPV16 and HPV31.

**Conclusions:**

This study lends support for HPV viral load and CpG methylation status, but not integration status, to be considered as potential biomarkers of cervical disease.

## Background

Persistent infection with oncogenic genotypes of genital Human Papillomavirus (HPV) is associated with the development of cervical cancer, a significant cause of morbidity and mortality of women worldwide [1]. Precancerous cervical disease is classified by cytological (low [LSIL] or high grade [HSIL] squamous intraepithelial lesions) and histological stages (cervical intraepithelial neoplasia [CIN] grades 1 to 3). There are about a dozen HPV types associated with the development of cervical cancer [2], differing in their relative contributions to the prevalence of cervical disease [3]. Testing for the presence of oncogenic HPV DNA offers improved sensitivity, though lower specificity, than cytology alone [4] while the next generation of molecular tests, including those with limited genotyping capability, may improve upon this [5]. Other potential molecular markers of cervical disease include type-specific viral load (VL), integration of HPV DNA into the host genome and methylation of the HPV genome. An improved understanding of the role of these potential molecular markers in cervical disease development may shed some light on HPV pathogenesis and may be helpful to guide future cervical cancer screening or treatment algorithms.

HPV DNA VL, usually estimated as the amount of HPV genome copies per cell, has been variably associated with cervical disease. Some studies were able to use HPV16 VL to differentiate between high grade (HSIL, CIN2+) and low grade (LSIL, CIN1) disease [6-8], between cervical cancer and lower grades of disease [9-11], or between any grade of cervical disease and normal samples [12]. Other studies could not find any association between HPV16 VL and cervical disease [13,14]. There are few studies examining any potential link between VL and cervical disease for other HPV types with some finding a positive association between some stages of disease for HPV18 [8,9], HPV31 [8], HPV33 [8], and HPV52 [9] while others have not [9,14-17].

HPV16 integration status has been able to distinguish between HSIL and LSIL samples [6], between cervical cancer samples and those of a lower grade of disease [9,10], or in some studies a strong positive correlation with increasing disease severity has been found [18]. In other studies no relationship between cervical disease grade and HPV16 integration status was apparent [11-13,19]. For HPV18 there are fewer studies overall with some finding an association with disease [9,18] and some not [16]. One study [18], found a strong positive correlation with increasing disease severity for HPV31, HPV33 and HPV45 while not for HPV52 [9,15] and HPV58 [9,17] in others.

Methylation of CpG sites within L1 [20-22], the upstream regulatory region (URR) [20,21,23] and/or other regions of the HPV16 genome [24-26] have often, but not always [27,28], been associated with cervical disease. Data on the degree of CpG site methylation for genotypes HPV18 [25,29,30], HPV31 [29] and HPV45 [29] are limited but appear to show a similar trend, suggesting that HPV methylation may be useful as a potential marker for cervical disease [31].

Some studies have examined both VL and integration status for HPV16 [6,7,9-13] but for other types including HPV18 [9,16], HPV52 [9,15] and HPV58 [9,17] the sources are limited. The VL of samples containing fully integrated HPV tends to be lower than that found in samples containing purely episomal or mixed forms [6,7,10,15], although this does not always appear to be the case [16,17]. Fewer studies have examined methylation status in relation to other parameters and then only for HPV16 infection [22,32].

Mixed infections are common throughout the course of cervical disease [3]. Few of these studies have explicitly used, or separately analyzed, samples harboring a single HPV type wherein the association between the HPV type under evaluation and cervical disease can be made with some confidence. Within these limited number of studies, the VL of samples harboring single infections has been associated with disease severity in some [7] but not in other studies [13,14,17]. For integration-based studies that explicitly mentioned the use of single infection samples, one study found an association between HPV16 integration status and disease [7] while another did not [13]. The only study explicitly to examine methylation levels in single infections (HPV18 and HPV31) demonstrated that some CpG sites exhibited higher methylation levels in CIN3 cases harboring single infections than in CIN3 cases with multiple infections with these types [29].

In this study, we evaluate the DNA viral load, integration and CpG methylation status of women singly infected with HPV16, HPV18, HPV31 or HPV45 in order better to understand the potential role for these molecular markers in cervical disease.

## Methods

### Samples

The present study made use of DNA (archived at -25°C for 2-3 years) from individuals singly infected with HPV16 (n = 226), HPV18 (n = 32), HPV31 (n = 75) or HPV45 (n = 29) from a cohort of 4,719 women attending cervical screening in England [33]. The age distributions within these monospecific infection groups were similar for HPV16 (median 39 [inter-quartile range, IQR, 31 - 48] years), HPV18 (42 [32 - 51]), HPV31 (41 [33 - 51]) and HPV45 (40 [26 - 43]) (*p* = 0.222 Kruskal-Wallis test). Accompanying histological data were available for ca. 15% of the cytology samples in the total study cohort. Amongst these, CIN2+ was diagnosed in 18% of borderline or mild dyskaryosis and in 79% of moderate or severe dyskaryosis. For analytical purposes, cytology grades of borderline or mild dyskaryosis were categorized as low grade (LG) cytology and cytology grades of moderate or severe dyskaryosis as high grade (HG) cytology. The testing of residual, anonymized DNA extracts for the purposes of improved understanding of cervical disease was approved by the Harrow Research Ethics Committee, UK (08/H0719/17).

Cell lines C33A (HTB-31, HPV negative), CaSki (CRL-1550; high copy HPV16), SiHa (HTB-35; low copy HPV16) and HeLa (CCL-2; HPV18) were from the American Type Culture Collection (LGC Standards, UK). Full genome plasmids were kindly provided by the German Cancer Research Centre (E.M. de Villiers: HPV16, HPV18, HPV45) and Qiagen Gaithersburg Inc., USA (A. Lorincz; HPV31). Human glyceraldehyde 3-phosphate dehydrogenase (GAPDH) and HPV E6 plasmids representing each type were made by insertion of PCR product into pCR2.1-TOPO (Invitrogen). The indicated reference sequences for HPV16 (K02718), HPV18 (X05015), HPV31 (J04353) and HPV45 (X74479) were used (http://pave.niaid.nih.gov).

### DNA viral load

PCR primers and probes targeting HPV E6 and GAPDH (Additional file 1: Table S1) were optimized for the ABI 7500 Fast PCR machine (Applied Biosystems) using Platinum UDG Supermix (Life Technologies). Full genome plasmid standards from 10^6^ - 10^1^ copies per reaction yielded median amplification efficiencies, linearity (*r*^*2*^) and the CV% of inter-assay C_T_ values of 96% (inter-quartile range, IQR, 92 – 98%), 0.998 (0.996 – 0.998), 3.1% (2.0 – 5.2%), respectively. VL is presented as HPV copies per cell (c/c) as determined by the viral copies per reaction/(GAPDH/2) copies per reaction. A positive control of pooled HPV16, HPV18, HPV31 and HPV45 DNA from a mixture of samples demonstrated good reproducibility: HPV16 median VL 0.39 c/c (IQR 0.32 – 0.61; n = 24), HPV18 1.66 c/c (1.50 – 1.88; n = 6); HPV31 0.22 c/c (0.14 – 0.25; n = 9) and HPV45 0.22 c/c (0.20 – 0.26; n = 9).

### Viral integration

The integration status of HPV16 [13], HPV18 [16], HPV31 and HPV45 was assessed using a ratio of the E2 gene copies over the E6 gene copies per reaction. Two estimates were made using an amino-terminal (Nt) and a carboxy-terminal (Ct) E2 PCR (Additional file 1: Table S1). PCR amplification was carried out on the ABI 7500 Fast PCR platform using Platinum SYBR green qPCR SuperMix-UDG (Life Technologies). Discriminatory power was determined using genome (representing episomal DNA) and E6 only (representing integrated DNA) plasmids in a background of C33A cells to simulate integration proportions of 0, 20, 50, 80, and 100% for each type. Linearity of these calibrators was good (median *r*^*2*^ 0.998 [IQR 0.994 – 0.998]). The lower limit of the 99% confidence interval (CI) for E2/E6 ratios obtained using the full length plasmid (0% integration) could be differentiated from the upper 99% CI of the E2/E6 ratios for samples containing a simulated integration level of 20% (*p* ≤ 0.01). This empirical threshold was used to differentiate between samples bearing fully episomal or mixed forms of the virus [7,11]. If the E2/E6 ratio for both Nt and Ct E2 fragments was above the respective empirical threshold, the sample was considered as bearing only episomal forms of the virus. If one or both fragments were not amplified, the sample was considered to bear fully integrated virus, otherwise the sample was designated as containing mixed forms of the virus.

### CpG methylation

The degree of CpG site-specific methylation within the 3’L1-URR regions of the HPV genome was estimated using methylation-specific PCR (Additional file 1: Table S1) of bisulfite-treated DNA (EZ DNA Methylation-Gold kit; Zymo Research) followed by pyrosequencing (HPV16) [34] or direct sequencing (HPV18, HPV31, HPV45) of five pCR2.1-TOPO clones per sample. The breadth and magnitude of CaSki, SiHa and HeLa cell CpG methylation were as expected [30,34]. CpG site methylation within the 3’L1-URR region of CaSki (n = 9 pCR2.1-TOPO clones) and SiHa (n = 10 clones) cells was similar to that obtained by pyrosequencing (Wilcoxon paired signed rank test, *p* = 0.400). Methylation levels of extracted SiHa DNA (n = 4) stored at -25°C for *ca*. 3 years were essentially the same as freshly cultured and extracted SiHa cells (n = 17; Pearson’s *r* =0.996; *p* < 0.001).

### Statistical analysis

The Mann Whitney *U* test and the test for trend were used to evaluate differences between two groups and three independent groups, respectively. Kruskal-Wallis was used to test for differences between multiple groups. The Wilcoxon paired sign rank test was used to test for differences between two groups of paired data.

Receiver operating characteristic (ROC) analyses were used to evaluate whether a parameter could differentiate between cases (HG with or without LG cytology samples) and controls (normal with or without LG cytology samples). The area under the curve (AUC) is a measure of how well a parameter could differentiate between these two groups (cases and controls), where no differentiation yields a value of 0.500 (equality) and scores of 0.800 or above are considered strong. The crossing point for the ROC curve was derived using the maximum Youden index (*J*) yielding the optimum balance of sensitivity and specificity and the resulting threshold of the parameter under study. The Fisher’s exact test was used to test for differences in proportions between cases and controls with crude odds ratios (95% CI) also given.

Tests were 2-tailed where appropriate and all tests were carried out using Stata 12.1 (StataCorp, USA).

## Results and discussion

### Viral load

**Figure 1:**
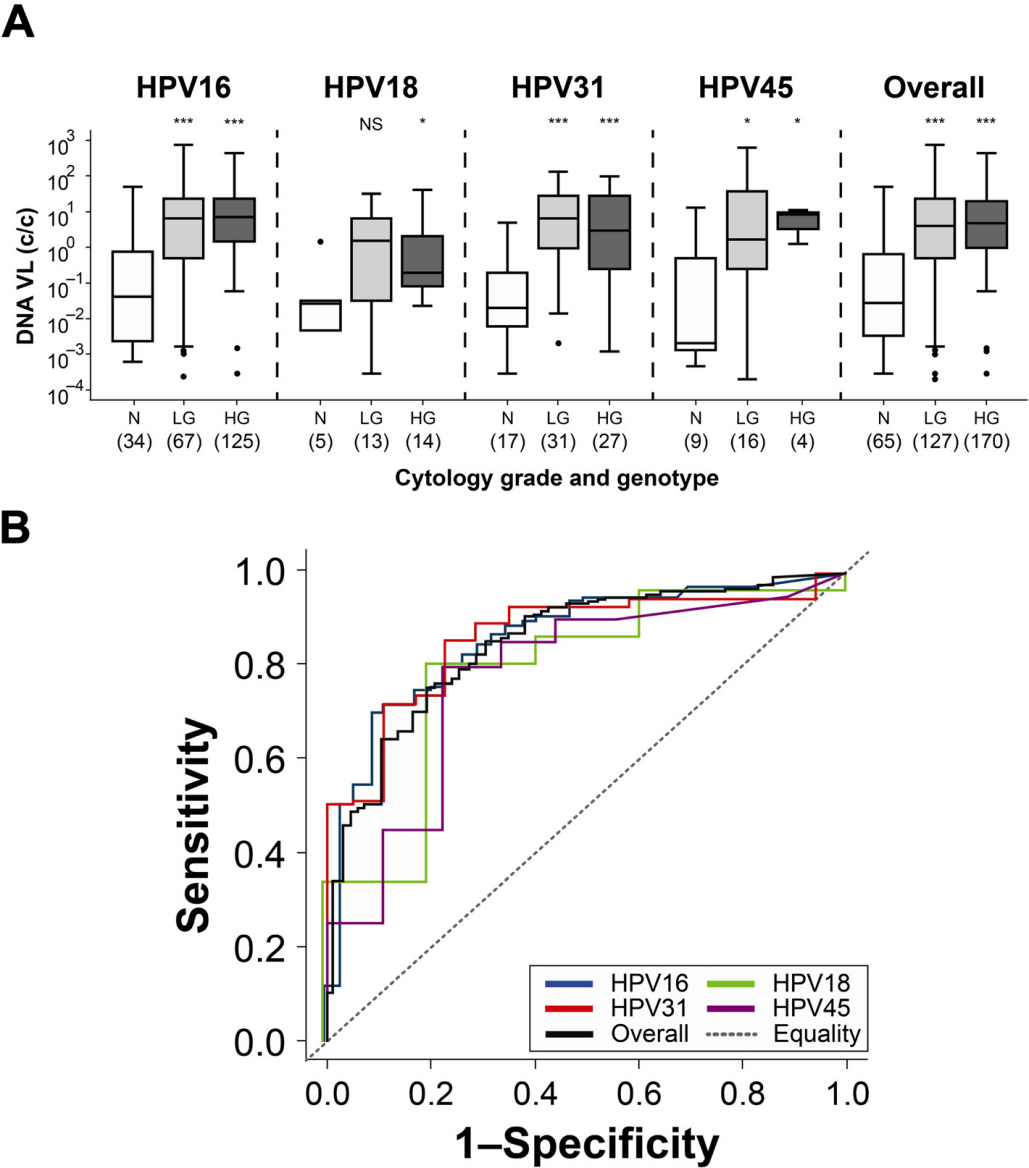
**Viral Load distribution according to disease grade and genotype. (A)** Box and whisker plots of individual and overall viral load segregated by samples exhibiting normal (white), low grade (light grey) or high grade (dark grey) cytology. Boxes represent the median and IQR, while the whiskers encompass 1.5 times the IQR. Outliers are represented by a black dot. NS, *p* > 0.05; *, *p* < 0.05; **, *p* < 0.01; ***, *p* < 0.001 (Mann Whitney *U* test between normal and LG or HG disease as indicated). **(B)** Receiver operator characteristic derived sensitivity and specificity plots for individual and overall viral load data. The line of equality represents an area under the curve (AUC) of 0.5.

The distribution of HPV16, HPV18, HPV31 and HPV45 DNA VL by cytology grade is shown in Figure 1A. The median HPV16 VL in normal cytology samples (0.04 c/c [IQR <0.01 – 0.71]; n = 34) was lower than that for low grade (LG) (6.59 c/c [0.50 – 22.72]; n = 67; *p* < 0.001; Mann Whitney *U* test) or high grade (HG) cytology samples (6.87 c/c [1.42 – 23.65]; n = 125; *p* < 0.001). The median HPV18 VL in normal cytology samples (0.03 c/c [<0.01 – 0.03]; n = 5) was also generally lower than the LG (1.35 c/c [0.03 – 6.08]; n = 13; *p* = 0.084) and HG samples (0.20 c/c [0.08 – 1.70]; n = 14; *p* = 0.033). This was also the case for HPV31 and HPV45. Thus, for all samples, regardless of HPV genotype, the median VL of normal cytology samples (0.03 c/c [<0.01 – 0.64]; n = 65) was lower than for the LG (4.01 c/c [0.51 – 22.72]; n = 127; *p* < 0.001) and HG cytology samples (4.62 c/c [0.98 – 20.00]; n = 170; *p* < 0.001).

**Figure 2:**
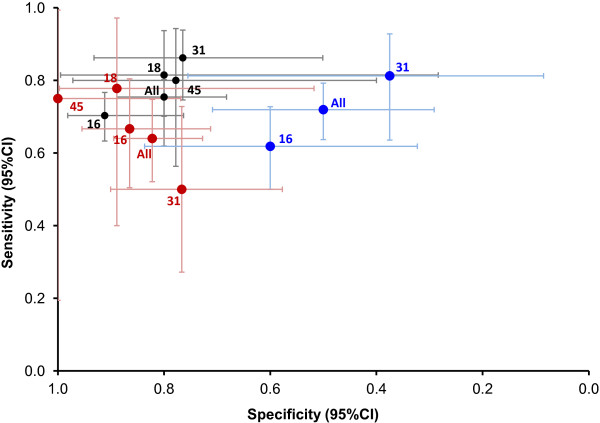
**Sensitivity and specificity plot.** Sensitivity and specificity plot with 95% CI shown for viral load (black), integration (blue) and methylation (red) status. Viral load and integration status were used to differentiate between normal and abnormal (LG and HG) cytology, whilst methylation status was used to differentiate normal and LG from HG samples.

We next wished to evaluate whether VL could differentiate between normal cytology and cervical disease with an appropriate degree of sensitivity and specificity. ROC plots for normal (control) versus LG and HG cytology (cases) samples are shown in Figure 1B while the resulting sensitivity and specificity plots for detection of disease are shown in Figure 2. HPV16 VL was able to distinguish between cases and controls with a sensitivity of 70% and a specificity of 91% (AUC 0.859) at a VL threshold of 1.58 c/c (OR 24.5 [95% CI 7.1 – 128.2]; *p* < 0.001). HPV18 VL was able to distinguish between cases and controls with a sensitivity of 81% and a specificity of 80% (AUC 0.800) at a VL threshold of 0.04 (OR 17.6 [1.2 – 904.3]; *p* = 0.015). HPV31 VL was able to distinguish between cases and controls with a sensitivity of 86% and a specificity of 78% (AUC 0.862) at a VL threshold of 0.22 (OR 20.3 [4.5 – 101.8]; *p* < 0.001). HPV45 VL was able to distinguish between cases and controls with a sensitivity of 80% and specificity of 78% (AUC 0.778) at a VL threshold of 0.51 (OR 12.0 [1.6 – 99.0]; *p* = 0.005). HPV VL overall was able to distinguish between cases and controls with a sensitivity of 75% and a specificity of 80% (AUC 0.844) at a VL threshold of 0.73 (OR 12.4 [6.2 – 26.1]; *p* < 0.001).

These data lend support to the potential for VL to be used to differentiate between normal and cytologically abnormal samples for a range of HPV types. Most other studies either did not include samples displaying normal cytology or histology [7-9], or could not make such a determination when they did [6,11,13-17]. Where ROC or similar analyses have been applied, two studies found that HPV16 VL could differentiate between HSIL and LSIL with a sensitivity of 50% and a specificity of 90% [6,7]. Another study found that VL levels could differentiate between low grade CIN and CIN2+ with improved sensitivity compared with cytology alone, but with an unacceptable impact on specificity [8]. Yet another study was able to differentiate between cervical cancer samples and those exhibiting CIN2 or CIN3 disease using HPV16, HPV18 and HPV52 (but not HPV58) VL levels [9]. It is not clear why there are such conflicting reports on the potential for an association between HPV VL and cervical disease, but the use of single infection samples may have helped in this respect.

### Viral integration

**Figure 3:**
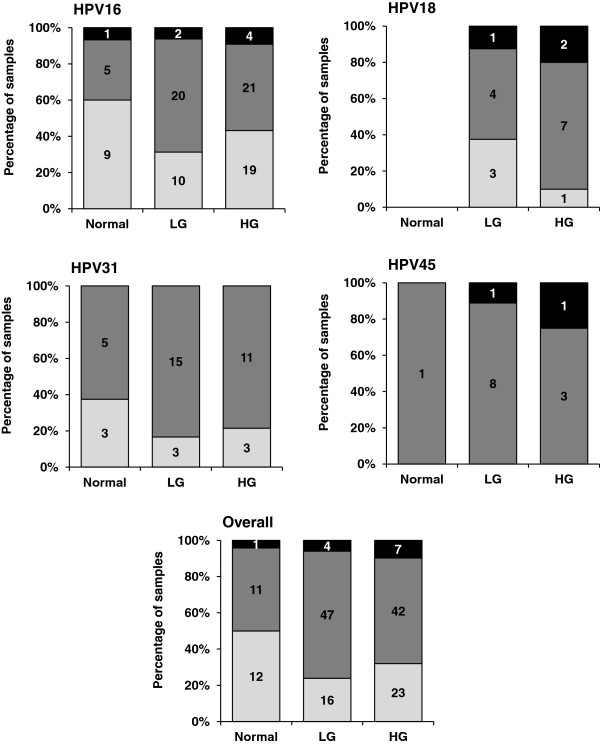
**Integration status distribution according to disease grade and genotype.** Number and percentage of normal, LG or HG cytology samples exhibiting episomal (white boxes), mixed (grey) or fully integrated (black) HPV genomes for each HPV type.

A comparison of the physical status of the HPV genome with cytological grade is shown in Figure 3. While a small proportion of samples harbored fully integrated HPV (n = 12 of 163 samples, 7.4%), the majority contained either a mixture of integrated and episomal forms or purely episomal forms. Of the fully integrated genomes (n = 12), samples lacking only the Ct E2 fragment or lacking both the Nt and Ct E2 fragments were more common (n = 4 [33%] and n = 7 [58%], respectively) than was disruption of the Nt E2 fragment alone (n = 1; 8%). Where this has been examined, studies report that disruption is commonly found in the Nt fragment of the E2 gene and entire E2 deletion is rare [13,15,17,35].

ROC analyses for the Nt E2/E6 and Ct E2/E6 ratios for HPV16 showed poor discrimination (AUC 0.430 and 0.415, respectively). The proportion of LG or HG cytology samples containing mixed or fully integrated forms was similar to those of the normal samples for HPV16 (OR 2.4 [95% CI 0.7 – 9.1]; *p* = 0.154) and HPV31 (OR 2.6 [0.3 – 18.0]; *p* = 0.348). For HPV18 and HPV45 there were too few normal samples to perform an appropriate analysis. For all samples, regardless of HPV type, there was a tendency for a slightly higher proportion of samples containing mixed or fully integrated forms in the cases (LG and HG samples) than in the controls (normal samples) as suggested by the OR of 2.6 (95% CI 1.0 – 6.8; *p* = 0.054) accompanied by a sensitivity of 72% but a poor specificity of 50% (Figure 2).

We used an empirical threshold [7,11] since in reconstitution experiments we could distinguish between simulated 0% and 20% integration levels. However, we also examined the potential for an association between integration status and disease using a range of thresholds (1.0, 0.8 and 0.5) for comparison with other studies [6,9,15-17,19]. Reducing the threshold had the effect of reducing the number of mixed forms leading to a decrease in the sensitivity and increase in the specificity for detection of LG or HG disease, although not reaching significance at any threshold (Additional file 2: Figure S1).

Although some studies could differentiate between cervical disease stages, usually between LG and HG disease using integration status [6,7,9], many could not [11-13,15-17,19]. Despite using samples harboring only single HPV types, where potentially confounding effects of targeting a non-disease associated type in a mixed infection sample should have been reduced, these data suggest that HPV integration status, at least as measured by E2/E6 PCR, may not be appropriately sensitive for utility as a diagnostic tool for cervical disease. Other assays such as APOT and/or DIPS may have better utility in this regard [18].

### CpG methylation

**Figure 4:**
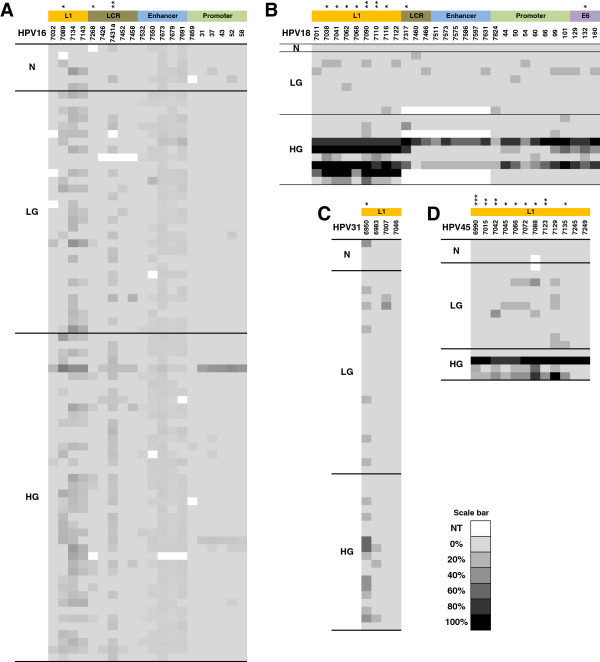
**Methylation status distribution according to disease grade and genotype.** Methylation profiles for **(A)** HPV16, **(B)** HPV18, **(C)** HPV31 and **(D)** HPV45 with each CpG site listed vertically across the top of each profile alongside their approximate genome position. The level of site-specific methylation is shaded according the scale bar from 0% - 100%. NT, a site did not exist in the given sample or failed to amplify. *, *p* < 0.05, **, *p* < 0.01, ***, *p* < 0.001 represent the significance level following a test for differences between normal and LG samples and HG samples (Mann Whitney *U* test).

The methylation status of CpG sites within the 3’L1-URR fragment of HPV16 (n = 79 samples) and HPV18 (n = 18) and of sites within the 3’L1 fragment of HPV31 (n = 50) and HPV45 (n = 18) are shown in Figure 4.

There were multiple CpG sites within the 3’L1-URR fragment of HPV16 that displayed low levels of methylation across all grades of cytology. On an individual CpG site basis, there were no sites whose methylation levels were able to distinguish normal samples from LG and HG samples (*p* > 0.05). However, there were three sites able to separate HG samples from LG and normal samples: one site in L1 (bp 7089; *p* = 0.011) and two in the URR (7268, *p* = 0.021 and 7431a, *p* = 0.007) (Figure 4A). The average methylation of these three sites could distinguish normal and LG (controls) from HG (cases) cytology samples with a sensitivity of 67% and a specificity of 86% (AUC 0.768) at a threshold of 5.3% (OR 12.8 [95%CI 3.7 – 49.6]; *p* < 0.001) (Figure 2). CpG site methylation in the 3’L1-URR fragment of HPV18 was similarly low in the normal and LG samples, but considerably higher in some of the HG samples, typically those classified as being severely dyskaryotic (Figure 4B). HG cytology samples could be distinguished from normal and LG samples with a sensitivity of 78% and a specificity of 89% (AUC 0.901) at an average methylation threshold of 6.7% across the nine HG disease-associated sites (OR 28.0 [95%CI 1.5 – 1413.3; *p* = 0.015) (Figure 2).

Of the sites in the short 3’L1 fragment of HPV31, methylation of the CpG site 6950 (Figure 4C) was weakly able to distinguish normal and LG samples from HG samples with a sensitivity of 50% and a specificity of 77% (AUC 0.656) at a threshold of 20% (OR 3.3 [95% CI 0.8 – 13.3]; *p* = 0.07) (Figure 2). For HPV45 there were nine sites associated with HG disease (Figure 4D) such that normal and LG samples could be distinguished from HG samples with a sensitivity of 75% and a specificity of 100% (AUC 0.839) at an average methylation threshold of 16.1% (*p* = 0.005) (Figure 2).

Overall, normal and LG samples could be distinguished from HG samples with a sensitivity of 64% and a specificity of 82% (AUC 0.746) at an average methylation level of 5.3% (OR 8.2 [95% CI 3.8 – 18.0; *p* < 0.001) (Figure 2). However, given the wide disparity in breadth and magnitude of methylated CpG sites between HPV genotypes, it may not be helpful to make such a generalization and perhaps highlights the need to treat each genotype individually.

These observations corroborate and extend previous reports on HPV16 [20,21,25] and HPV18 [25,29,30] demonstrating that while some CpG sites in HPV16 may be weakly associated with increasing cervical disease severity, HPV18-associated high grade cervical disease is characterized by broad CpG site methylation of a high magnitude. Two recent studies suggest that the methylation status of certain key HPV16 CpG sites may be able to differentiate between cervical cancer and low grade disease [22,36] and may even have the utility to be predictive [22]. Data on the methylation profiles of HPV31 and HPV45 are limited [29,37]. One study demonstrated differences between CIN3 cases and asymptomatic controls across a range of sites [29], while the other reported the proportion, but not the magnitude, of methylated sites in the HPV genome and a cellular gene increased with increasing disease severity [37]. The present data appear to suggest that this highly methylated state probably only occurs late in disease. In addition, these data corroborate the finding that there is an apparent species-specific association in HPV genome methylation [29], given the quite different methylation profiles of HPV types within the alpha-9 (HPV16, HPV31) and alpha-7 (HPV18, HPV45) species groups.

### Association between molecular parameters

Samples harboring solely integrated HPV16 DNA had a lower VL (median 0.14 c/c [IQR 0.11 – 0.30]; n = 7) than samples with solely episomal (1.26 c/c [0.71 – 21.25]; n = 38; *p* = 0.007) or mixed (0.99 c/c [0.43 – 22.64]; n = 46; *p* = 0.008) forms. For HPV31 samples containing mixed forms had a higher VL (4.40 c/c [0.76 – 7.55]; n = 31) than those with purely episomal forms (0.44 c/c [0.15 – 0.67]; n = 9; *p* = 0.001). For HPV18, solely integrated HPV18 DNA had a lower median VL (0.04 c/c [0.04 – 0.06]; n = 3) than samples with solely episomal (0.23 [0.07 – 0.61]; n = 4; *p* = 0.157) or mixed (2.87 [0.20 – 5.23]; n = 11; *p* = 0.073) forms. For HPV45 there were too few samples containing solely episomal or integrated forms to carry out this assessment. Overall, samples with solely integrated HPV DNA tended to have a lower VL (0.11 c/c [0.06 – 0.23]; n = 12) than samples with solely episomal (1.00 c/c [0.38 – 7.85]; n = 51; *p* = 0.003) or mixed (1.38 c/c [0.53 – 8.23]; n = 100; *p* = 0.001) forms while samples containing episomal or mixed forms had similar VL (*p* = 0.238). These data support the observation that samples harboring fully integrated HPV DNA tend to have lower VL than samples containing episomal or mixed forms [6,7,10,15] in contrast to other studies [16,17,38].

The VL in samples exhibiting one or more methylated CpG sites in the 3’L1 fragment of HPV16 (median VL 27.81 c/c [IQR 12.75 – 64.55]; n = 60) was similar to those samples that had no methylated CpG sites (18.86 c/c [5.80 – 45.09]; n = 19; *p* = 0.180). This was also the case if the VL values were segregated according to whether disease-associated CpG sites (7089, 7268 and 7431a) were methylated (VL 30.17 c/c [10.29 – 62.71]; n = 61) or not (21.52 c/c [11.58 – 47.08]; n = 61; *p* = 0.752). For HPV31 samples exhibiting one or more methylated CpG sites in the 3’L1 fragment tended to have a lower median VL (1.12 c/c [0.54 – 3.78]; n = 19) than samples exhibiting unmethylated CpG sites (8.83 c/c [4.50 – 33.01]; n = 31; *p* < 0.001). The VL of samples exhibiting methylated (1.62 c/c [0.57 – 3.84]; n = 12) or unmethylated CpG sites (5.62 c/c [2.39 – 25.37]; n = 6; *p* = 0.134) in the 3’L1 region for HPV18 were similar, as were the VL of samples exhibiting methylated (9.31 c/c [1.26 – 30.71]; n = 9) or unmethylated (4.51 c/c [1.92 – 11.91]; n = 9; *p* = 0.627) sites in the 3’L1 fragment of HPV45.

There was no difference between the average 3’L1 methylation levels for those samples containing episomal (7.27% [95% CI 5.05 – 10.45]; n = 16) and those with mixed (7.60% [5.6 – 10.28]; n = 33) forms (*p* = 0.691). The lack of association may be confounded by having too few fully integrated samples to include in the analysis and/or the apparently poor precision of the E2/E6 PCR to determine the integration status.

## Conclusions

The purpose of this study was to evaluate any potential relationship between HPV type-specific viral load, integration and methylation status and current cervical disease stage with regard to a single infecting HPV genotype, wherein the association with disease can be assumed with some confidence.

There are potential shortcomings to this study. First, samples containing a single infecting genotype were identified using a generic PCR and genotyping test [33]. While this is the most common approach used and the test used arguably one of the more robust [39], such a determination is not without its problems including the potential for masking within mixed infections [40]. The selection of single infection samples is, we believe, a significant improvement on the use of unselected samples, wherein the potential impact of such masking is likely to be far higher. Nevertheless, the possibility that a minority of samples in this study contained low levels of one or more other HPV types cannot be ruled out. Second, although these samples constitute a highly selected panel and a minority sample type collected during routine screening, they do nevertheless permit the evaluation of the potential for these markers to be used, with a limited number of other confounding factors. Finally, although cytology samples are the primary sample type collected during cervical screening and are readily amenable to such testing, the use of cytology samples alone to improve the definition of cervical disease stage is problematic given the often discontinuous relationship between cytology and histology stage designations [1].

In summary, even under these optimized conditions, the sensitivities and specificities of many of these potential molecular markers were lower than the median sensitivity (98%) and specificity (86%) obtained in a recent evaluation of commercial HPV DNA tests for identifying CIN2+ cases during cytological screening [5]. There were a few individual measurements that have the potential for use in triage, characterized by a similar or higher specificity than required for initial screening. HPV VL appears to have an overall specificity of *ca*. 80% (range 76 - 91%) to differentiate normal cytology from abnormal cytology. Conversely, HPV methylation appears to be able to differentiate HG cytology from normal and LG cytology with a specificity of 77 - 100%. Both of these measures, therefore, have potential for use in screening and/or triage, but the utility of HPV genome methylation status may be improved if the disease-associated genotype-specific CpG site methylation patterns can be appropriately exploited.

## Competing interests

The authors declare no conflicts of interest.

## Authors’ contributions

LM conceived and designed the experiments, performed the experiments, analyzed the data and contributed to drafting the manuscript. AG conceived and designed the experiments, analyzed the data and contributed to drafting the manuscript. SB conceived and designed the experiments, analyzed the data and contributed to drafting the manuscript. JVP contributed to drafting the manuscript. All authors read and approved the final manuscript.

## Pre-publication history

The pre-publication history for this paper can be accessed here:

http://www.biomedcentral.com/1471-2407/14/384/prepub

## Supplementary Material

Additional file 1: Table S1 Primer and Probes. Click here for file

Additional file 2: Figure S1 Impact of Varying Integration Thresholds. Top panels depict the number and percentage of samples exhibiting episomal, mixed or fully integrated genomes arsing from the use of a range of E2/E6 thresholds. Bottom panels depict the sensitivity and specificity plots for integration status being able to differentiate between normal and abnormal cytology (LG and HG) at a range of E2/E6 thresholds. *p* values refer to the proportion of mixed or fully integrated samples compared to episomal samples when using all samples regardless of HPV type (Fisher’s exact test). Click here for file

## References

[B1] Schiffman M Castle PE Jeronimo J Rodriguez AC Wacholder S Human papillomavirus and cervical cancer Lancet 2007 370 890 907 10.1016/S0140-6736(07)61416-0 17826171

[B2] Bouvard V Baan R Straif K Grosse Y Secretan B El Ghissassi F Benbrahim-Tallaa L Guha N Freeman C Galichet L Cogliano V A review of human carcinogens--Part B: biological agents Lancet Oncol 2009 10 321 322 10.1016/S1470-2045(09)70096-8 19350698

[B3] Guan P Howell-Jones R Li N Bruni L de Sanjose S Franceschi S Clifford GM Human papillomavirus types in 115,789 HPV-positive women: a meta-analysis from cervical infection to cancer Int J Canc 2012 131 2349 2359 10.1002/ijc.27485 22323075

[B4] Cuzick J Clavel C Petry KU Meijer CJ Hoyer H Ratnam S Szarewski A Birembaut P Kulasingam S Sasieni P Iftner T Overview of the European and North American studies on HPV testing in primary cervical cancer screening Int J Canc 2006 119 1095 1101 10.1002/ijc.21955 16586444

[B5] Cuzick J Cadman L Mesher D Austin J Ashdown-Barr L Ho L Terry G Liddle S Wright C Lyons D Szarewski A Comparing the performance of six human papillomavirus tests in a screening population Br J Canc 2013 108 908 913 10.1038/bjc.2013.22 PMC3590662 23370211

[B6] Saunier M Monnier-Benoit S Mauny F Dalstein V Briolat J Riethmuller D Kantelip B Schwarz E Mougin C Pretet JL Analysis of human papillomavirus type 16 (HPV16) DNA load and physical state for identification of HPV16-infected women with high-grade lesions or cervical carcinoma J Clin Microbiol 2008 46 3678 3685 10.1128/JCM.01212-08 18799702 PMC2576617

[B7] Cricca M Morselli-Labate AM Venturoli S Ambretti S Gentilomi GA Gallinella G Costa S Musiani M Zerbini M Viral DNA load, physical status and E2/E6 ratio as markers to grade HPV16 positive women for high-grade cervical lesions Gynecol Oncol 2007 106 549 557 10.1016/j.ygyno.2007.05.004 17568661

[B8] Hesselink AT Berkhof J Heideman DA Bulkmans NW van Tellingen JE Meijer CJ Snijders PJ High-risk human papillomavirus DNA load in a population-based cervical screening cohort in relation to the detection of high-grade cervical intraepithelial neoplasia and cervical cancer Int J Canc 2009 124 381 386 10.1002/ijc.23940 19003961

[B9] Ho CM Chien TY Huang SH Lee BH Chang SF Integrated human papillomavirus types 52 and 58 are infrequently found in cervical cancer, and high viral loads predict risk of cervical cancer Gynecol Oncol 2006 102 54 60 10.1016/j.ygyno.2005.11.035 16386784

[B10] Das D Bhattacharjee B Sen S Mukhopadhyay I Sengupta S Association of viral load with HPV16 positive cervical cancer pathogenesis: causal relevance in isolates harboring intact viral E2 gene Virology 2010 402 197 202 10.1016/j.virol.2010.03.030 20394955

[B11] Boulet GA Benoy IH Depuydt CE Horvath CA Aerts M Hens N Vereecken AJ Bogers JJ Human papillomavirus 16 load and E2/E6 ratio in HPV16-positive women: biomarkers for cervical intraepithelial neoplasia >or=2 in a liquid-based cytology setting? Canc Epidemiol Biomarkers Prev 2009 18 2992 2999 10.1158/1055-9965.EPI-09-0025 19861526

[B12] Briolat J Dalstein V Saunier M Joseph K Caudroy S Pretet JL Birembaut P Clavel C HPV prevalence, viral load and physical state of HPV-16 in cervical smears of patients with different grades of CIN Int J Canc 2007 121 2198 2204 10.1002/ijc.22959 17657742

[B13] Cheung JL Lo KW Cheung TH Tang JW Chan PK Viral load, E2 gene disruption status, and lineage of human papillomavirus type 16 infection in cervical neoplasia J Infect Dis 2006 194 1706 1712 10.1086/509622 17109343

[B14] Flores R Papenfuss M Klimecki WT Giuliano AR Cross-sectional analysis of oncogenic HPV viral load and cervical intraepithelial neoplasia Int J Canc 2006 118 1187 1193 10.1002/ijc.21477 16152619

[B15] Cheung JL Cheung TH Tang JW Chan PK Increase of integration events and infection loads of human papillomavirus type 52 with lesion severity from low-grade cervical lesion to invasive cancer J Clin Microbiol 2008 46 1356 1362 10.1128/JCM.01785-07 18272718 PMC2292946

[B16] Cheung JL Cheung TH Ng CW Yu MY Wong MC Siu SS Yim SF Chan PK Analysis of human papillomavirus type 18 load and integration status from low-grade cervical lesion to invasive cervical cancer J Clin Microbiol 2009 47 287 293 10.1128/JCM.01531-08 19036939 PMC2643667

[B17] Chan PK Cheung JL Cheung TH Lo KW Yim SF Siu SS Tang JW Profile of viral load, integration, and E2 gene disruption of HPV58 in normal cervix and cervical neoplasia J Infect Dis 2007 196 868 875 10.1086/520884 17703417

[B18] Vinokurova S Wentzensen N Kraus I Klaes R Driesch C Melsheimer P Kisseljov F Durst M Schneider A von Knebel Doeberitz M Type-dependent integration frequency of human papillomavirus genomes in cervical lesions Canc Res 2008 68 307 313 10.1158/0008-5472.CAN-07-2754 18172324

[B19] Kulmala SM Syrjanen SM Gyllensten UB Shabalova IP Petrovichev N Tosi P Syrjanen KJ Johansson BC Early integration of high copy HPV16 detectable in women with normal and low grade cervical cytology and histology J Clin Pathol 2006 59 513 517 10.1136/jcp.2004.024570 16484445 PMC1860285

[B20] Kalantari M Calleja-Macias IE Tewari D Hagmar B Lie K Barrera-Saldana HA Wiley DJ Bernard HU Conserved methylation patterns of human papillomavirus type 16 DNA in asymptomatic infection and cervical neoplasia J Virol 2004 78 12762 12772 10.1128/JVI.78.23.12762-12772.2004 15542628 PMC525027

[B21] Sun C Reimers LL Burk RD Methylation of HPV16 genome CpG sites is associated with cervix precancer and cancer Gynecol Oncol 2011 121 59 63 10.1016/j.ygyno.2011.01.013 21306759 PMC3062667

[B22] Mirabello L Schiffman M Ghosh A Rodriguez AC Vasiljevic N Wentzensen N Herrero R Hildesheim A Wacholder S Scibior-Bentkowska D Burk RD Lorincz AT Elevated methylation of HPV16 DNA is associated with the development of high grade cervical intraepithelial neoplasia Int J Canc 2013 132 1412 1422 10.1002/ijc.27750 PMC3493709 22847263

[B23] Snellenberg S Schutze DM Claassen-Kramer D Meijer CJ Snijders PJ Steenbergen RD Methylation status of the E2 binding sites of HPV16 in cervical lesions determined with the Luminex(R) xMAP system Virology 2012 422 357 365 10.1016/j.virol.2011.11.006 22137333

[B24] Brandsma JL Sun Y Lizardi PM Tuck DP Zelterman D Haines GK 3rd Martel M Harigopal M Schofield K Neapolitano M Distinct human papillomavirus type 16 methylomes in cervical cells at different stages of premalignancy Virology 2009 389 100 107 10.1016/j.virol.2009.03.029 19443004 PMC2918277

[B25] Fernandez AF Rosales C Lopez-Nieva P Grana O Ballestar E Ropero S Espada J Melo SA Lujambio A Fraga MF Pino I Javierre B Carmona FJ Acquadro F Steenbergen RD Snijders PJ Meijer CJ Pineau P Dejean A Lloveras B Capella G Quer J Buti M Esteban JI Allende H Rodriguez-Frias F Castellsague X Minarovits J Ponce J Capello D The dynamic DNA methylomes of double-stranded DNA viruses associated with human cancer Genome Res 2009 19 438 451 19208682 10.1101/gr.083550.108 PMC2661803

[B26] Mirabello L Sun C Ghosh A Rodriguez AC Schiffman M Wentzensen N Hildesheim A Herrero R Wacholder S Lorincz A Burk RD Methylation of human papillomavirus type 16 genome and risk of cervical precancer in a Costa Rican population J Natl Canc Inst 2012 104 556 565 10.1093/jnci/djs135 PMC3317880 22448030

[B27] Xi LF Jiang M Shen Z Hulbert A Zhou XH Lin YY Kiviat NB Koutsky LA Inverse association between methylation of human papillomavirus type 16 DNA and risk of cervical intraepithelial neoplasia grades 2 or 3 PLoS One 2011 6 e23897 10.1371/journal.pone.0023897 21887341 PMC3161083

[B28] Badal V Chuang LS Tan EH Badal S Villa LL Wheeler CM Li BF Bernard HU CpG methylation of human papillomavirus type 16 DNA in cervical cancer cell lines and in clinical specimens: genomic hypomethylation correlates with carcinogenic progression J Virol 2003 77 6227 6234 10.1128/JVI.77.11.6227-6234.2003 12743279 PMC154984

[B29] Wentzensen N Sun C Ghosh A Kinney W Mirabello L Wacholder S Shaber R LaMere B Clarke M Lorincz AT Castle PE Schiffman M Burk RD Methylation of HPV18, HPV31, and HPV45 genomes and cervical intraepithelial neoplasia grade 3 J Natl Canc Inst 2012 104 1738 1749 10.1093/jnci/djs425 PMC3571257 23093560

[B30] Turan T Kalantari M Calleja-Macias IE Cubie HA Cuschieri K Villa LL Skomedal H Barrera-Saldana HA Bernard HU Methylation of the human papillomavirus-18 L1 gene: a biomarker of neoplastic progression? Virology 2006 349 175 183 10.1016/j.virol.2005.12.033 16472835

[B31] Clarke MA Wentzensen N Mirabello L Ghosh A Wacholder S Harari A Lorincz A Schiffman M Burk RD Human papillomavirus DNA methylation as a potential biomarker for cervical cancer Canc Epidemiol Biomarkers Prev 2012 21 2125 2137 10.1158/1055-9965.EPI-12-0905 PMC3664203 23035178

[B32] Das Ghosh D Bhattacharjee B Sen S Premi L Mukhopadhyay I Chowdhury RR Roy S Sengupta S Some novel insights on HPV16 related cervical cancer pathogenesis based on analyses of LCR methylation, viral load, E7 and E2/E4 expressions PLoS One 2012 7 e44678 10.1371/journal.pone.0044678 22970286 PMC3435323

[B33] Howell-Jones R Bailey A Beddows S Sargent A de Silva N Wilson G Anton J Nichols T Soldan K Kitchener HC Multi-site study of HPV type-specific prevalence in women with cervical cancer, intraepithelial neoplasia and normal cytology, in England Br J Canc 2010 103 209 216 10.1038/sj.bjc.6605747 PMC2906740 20628396

[B34] Rajeevan MS Swan DC Duncan K Lee DR Limor JR Unger ER Quantitation of site-specific HPV 16 DNA methylation by pyrosequencing J Virol Meth 2006 138 170 176 10.1016/j.jviromet.2006.08.012 17045346

[B35] Arias-Pulido H Peyton CL Joste NE Vargas H Wheeler CM Human papillomavirus type 16 integration in cervical carcinoma in situ and in invasive cervical cancer J Clin Microbiol 2006 44 1755 1762 10.1128/JCM.44.5.1755-1762.2006 16672403 PMC1479176

[B36] Bryant D Tristram A Liloglou T Hibbitts S Fiander A Powell N Quantitative measurement of Human Papillomavirus type 16 L1/L2 DNA methylation correlates with cervical disease grade J Clin Virol 2014 59 24 29 10.1016/j.jcv.2013.10.029 24268385

[B37] Kalantari M Osann K Calleja-Macias IE Kim S Yan B Jordan S Chase DM Tewari KS Bernard HU Methylation of human papillomavirus 16, 18, 31, and 45 L2 and L1 genes and the cellular DAPK gene: Considerations for use as biomarkers of the progression of cervical neoplasia Virology 2014 448 314 321 24314662 10.1016/j.virol.2013.10.032 PMC4051423

[B38] Cheung JL Cheung TH Yu MY Chan PK Virological characteristics of cervical cancers carrying pure episomal form of HPV16 genome Gynecol Oncol 2013 131 374 379 10.1016/j.ygyno.2013.08.026 24012799

[B39] Eklund C Forslund O Wallin KL Dillner J Global improvement in genotyping of Human Papillomavirus DNA: The 2011 HPV LabNet International Proficiency Study J Clin Microbiol 2014 52 449 459 10.1128/JCM.02453-13 24478473 PMC3911320

[B40] van Alewijk D Kleter B Vent M Delroisse JM de Koning M van Doorn LJ Quint W Colau B A human papilloma virus testing algorithm comprising a combination of the L1 broad-spectrum SPF10 PCR assay and a novel E6 high-risk multiplex type-specific genotyping PCR assay J Clin Microbiol 2013 51 1171 1178 10.1128/JCM.02831-12 23363835 PMC3666791

