# Supplementary material for: Human Papillomavirus 16, 18, 31 and 45 viral load, integration and methylation status stratified by cervical disease stage
Source: BMC Cancer. 2014 May 30;14:384. doi: 10.1186/1471-2407-14-384 (PMC4053304; doi:10.1186/1471-2407-14-384)
Supplement: Additional file 1: Table S1 — Primer and Probes. [file 1471-2407-14-384-S1.pdf]

Supplementary Table S1 – PCR primer and probe sequences

Parameter	Target	Region	Name	Location (5' - 3')	Sequence (5' - 3')	Size
DNA VL	HPV16	E6	E6 For (1)	382-407	CAAACCGTTGTGTGATTTGTTAATTA	89
			E6 Rev (1)	448-470	GCTTTTTGTCCAGATGTCTTTGC	
			E6 Probe (1)	410-445	TGTATTAACTGTCAAAAGCCACTGTGTCCTGAAGAA (7)	
	HPV18	E6	18 F (2)	507-525	AGAGGCCAGTGCCATTCGT	65
			18 R (2)	551-571	GTTTCTCTGCGTCGTTGGAGT	
			18 P (2)	526-547	TCCTGTCGTGCTCGGTTGCAGC (7)	
	HPV31	E6	31 F (2)	479-502	ATTCCACAACATAGGAGGAAGGTG	76
			31 R (2)	531-554	CACTTGGGTTTCAGTACGAGGTCT	
			31 P (2)	504-528	ACAGGACGTTGCATAGCATGTTGGA (7)	
	HPV45	E6	45 F (2)	495-517	GGACAGTACCGAGGGCAGTGTAA	71
			45 R (2)	543-565	TCCCTACGTCTGCGAAGTCTTTC	
			45 P (2)	520-541	CATGTTGTGACCAGGCACGGCA (7)	
	Cell	GAPDH	GAPDH forward (3)	2040-2921	GGACTGAGGCTCCCACCTTT	188
			GAPDH reverse (3)	3089-3108	GCATGGACTGTGGTCTGCAA	
			HT probe (3)	2943-2966	CATCCAAGACTGGCTCCTCCCTGC (7)	
Methylation	HPV16	PE	A FW (4)	7829-7851	TTGTAAAATTGTATATGGGTGTG (9)	193
			A REV (4)	94-117	AAATCCTAAAACATTACAATTCTC (8), (9)	
			400 S1 (4)	9-30	AATTTATGTATAAAATTAAGGG (10)	
			400 S2 (4)	7838-7858	TGTATATGGGTGTGTGTAAAT (10)	
		5’-LCR	B FW (4)	7459-4781	GGTTGTATGTTTTTTGGTATAAA (9)	263
			B REV (4)	7692-7721	AAAACAAACCAAAAATATATACCTAACAAC (8), (9)	
			500 S1 (4)	7510-7529	GTAATTATGGTTTAAATTTG (10)	
			500 S2 (4)	7645-7664	ATTGTGTTATATAAAATAAA (10)	
		5'-LCR	700 (4)	7211-7230	GTATGTGTTTGTAAATATTA (8), (9)	281
			C REV (4)	7491-7468	AAAAACACATTTTATACCAAAAAA (9)	
			600 (4)	7463-7482	TTTTATACCAAAAAACATAC (10)	
		3'-L1	D FW (4)	6974-6996	TTGGGAAGTAAATTTAAAGGAAA (9)	390
			D REV (4)	7340-7363	AATAACCACAACACAATTAATAAA (8), (9)	
			1000 (4)	7011-7030	TTAGATTAGTTTTTTTTAGG (10)	
		3'-L1	E FW (4)	6975-6994	TGGGAAGTAAATTTAAAGGA (8), (9)	386
			E REV (4)	7340-7360	AACCACAACACAATTAATAAA (9)	
			800 (4)	7147-7166	TACATACAATACTTACAACT (10)	
		3'-L1/5'-LCR	F FW (4)	7049-7072	AGTAGGATTGAAGGTTAAATTAAA (9)	315
			F REV (4)	7340-7363	AATAACCACAACACAATTAATAAA (8), (9)	
			700 (4)	7211-7230	GTATGTGTTTGTAAATATTA (10)	
			900 (4)	7067-7086	ATTAAAATTTATATTAGGAA (10)	
	HPV18	5’-LCR	Msp10F (5)	7280-7303	TAAAATATGTTTTGTGGTTTTGTG	468
			Msp10R (5)	7721-7747	ATAATTATACAAACCAAATATACAATT	
		P_E / 5’-E6	Msp8F (5)	7753-7781	TGTTTAATATTTTGTTTATTTTTAATATG	291
			Msp8R (5)	161-786	TATCTTACAATAAAATATTCAATTCC	
		3’-L1	18 E FWD (6)	6968-6997	AAAGGAAAAGTTTTTTTTAGATTTAGATTA	221
			18 E REV (6)	7163-7188	ACAAAACATACAAACACAACAATAAA	
	HPV31	3’-L1	31 L FWD (6)	6908-6935	AAAGAAAAGTTTTTTGTAGATTTAGATT	205
			31 L REV (6)	7087-7112	ACACAAACACATACACATACAAACAC	
	HPV45	3’-L1 5'-LCR	45 L FWD (6)	6954-6976	GTTTTGGATTGTTGATTTAAAGG	336
			45 L REV (6)	7264-7289	ATAACACCATACATACCACAAAACAC	
Integration	HPV16	E6	(1)	–	same for DNA VL, above	–
		E2-5’	16 E2-5 FWD (6)	2852-2873	GGAAACACATGCGCCTAGAATG	132
			16 E2-5 REV (6)	2959-2983	GTTGCAGTTCAATTGCTTGTAATGC	
		E2-3’	16 E2-3 FWD (6)	3683-3704	$^{C}/_{A}$ATTGTATACTGCAGTGTCGTC	104
			16 E2-3 REV (6)	3767-3786	GTCACGTTGCCATTCACTAT	
	HPV18	E6	(2)	–	same for DNA VL, above	–
		E2-5’	18 E2-5 FWD (6)	3109-3128	TGCAAGACACATGCGAGGAA	157
			18 E2-5 REV (6)	3244-3265	CATGTTCCTGCATCAGTCATAT	
		E2-3’	18 E2-3 FWD (6)	3632-3652	TACAGGCAACAACAAAAGACG	177
			18 E2-3 REV (6)	3789-3808	CCTGTTTTTTCATTGCCTGC	
	HPV31	E6	(2)	–	same for DNA VL, above	–
		E2-5’	31 E2-5 FWD (6)	2650-2671	TGGTGCAGATTAAATTTGCACG	63
			31 E2-5 REV (6)	2691-2712	CGTTGAGAAAGAGTCTCCATCG	
		E2-3’	31 E2-3 FWD (6)	3429-3448	CAAGAACAGAGCCAGAGCAC	78
			31 E2-3 REV (6)	3487-3506	AGTTGACACTGTCCACGGAG	
	HPV45	E6	(2)	–	same for DNA VL, above	–
		E2-5’	45 E2-5 FWD (6)	2752-2768	AGATTTGCACGAGGACG	39
			45 E2-5 REV (6)	2776-2790	CCTTCGGTGTCTGCA	
		E2-3’	45 E2-3 FWD (6)	3843-3860	GTACAAATCTCGGTGGGA	132
			45 E2-3 REV (6)	3960-3974	AAGCGGGACATTGCA	

(1) Oligonucleotide derived from the literature (Lanham et al., 2001).
(2) Oligonucleotide derived from the literature (Lindh et al., 2007).
(3) Oligonucleotide derived from the literature (Payan et al., 2007).
(4) Oligonucleotide derived from the literature (Rajeevan et al., 2006).
(5) Oligonucleotide derived from the literature (Turan et al., 2006).
(6) Oligonucleotide designed in house.
(7) Oligonucleotide modified as follows: 5'-FAM, 3'-TAMRA.
(8) Oligonucleotide modified as follows: 5'-biotin.
(9) Oligonucleotide used for amplification.
(10) Oligonucleotide used for pyrosequencing.
