# Supplementary figures and images for: Human Papillomavirus 16, 18, 31 and 45 viral load, integration and methylation status stratified by cervical disease stage
Source: BMC Cancer. 2014 May 30;14:384. doi: 10.1186/1471-2407-14-384 (PMC4053304; doi:10.1186/1471-2407-14-384)

Supplementary Figure S1

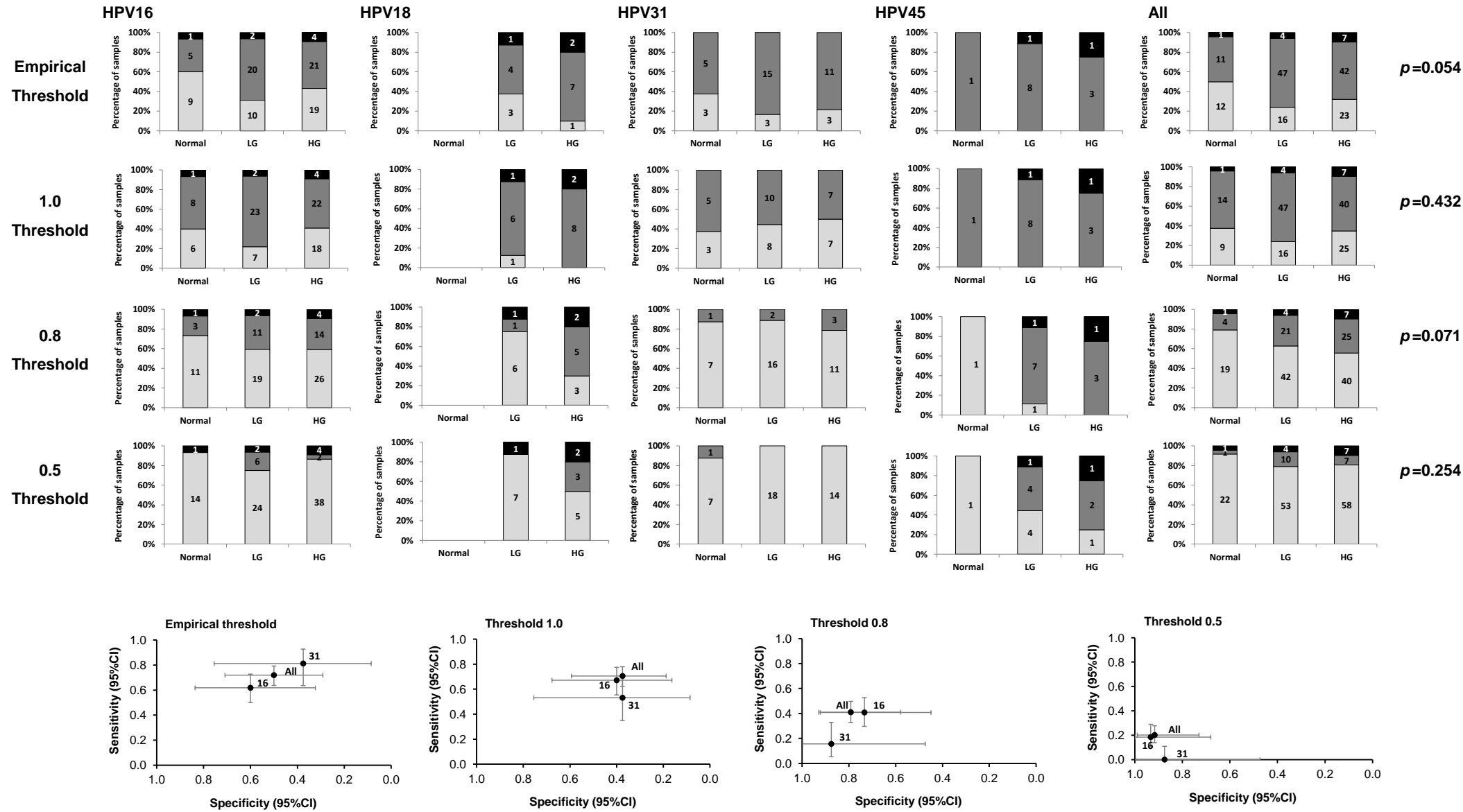

Supplement: Additional file 2: Figure S1 — Impact of Varying Integration Thresholds. Top panels depict the number and percentage of samples exhibiting episomal, mixed or fully integrated genomes arsing from the use of a range of E2/E6 thresholds. Bottom panels depict the sensitivity and specificity plots for integration status being able to differentiate between normal and abnormal cytology (LG and HG) at a range of E2/E6 thresholds. p values refer to the proportion of mixed or fully integrated samples compared to episomal samples when using all samples regardless of HPV type (Fisher’s exact test). [file 1471-2407-14-384-S2.pdf]
